# Impact of Button Mushroom Stem Residue as a Functional Ingredient for Improving Nutritional Characteristics of Pizza Dough

**DOI:** 10.3390/molecules29215140

**Published:** 2024-10-30

**Authors:** Miguel A. Gallardo, Wagner G. Vieira Júnior, María Esther Martínez-Navarro, Manuel Álvarez-Ortí, Diego C. Zied, José E. Pardo

**Affiliations:** 1 Escuela Técnica Superior de Ingeniería Agronómica y de Montes y Biotecnología, Campus Universitario s/n, 02071 Albacete, Spain; miguelangel.gallardo@alu.uclm.es (M.A.G.); mesther.martinez@uclm.es (M.E.M.-N.); manuel.alvarez@uclm.es (M.Á.-O.); 2 Programa de Pós-Graduação em Microbiologia Agropecuária, Faculdade de Ciências Agrárias e Veterinárias, Universidade Estadual Paulista (UNESP), Jaboticabal 14884-900, Brazil; vieira.jr@unesp.br (W.G.V.J.); diego.zied@unesp.br (D.C.Z.)

**Keywords:** mushroom residue, waste, food reuse, food enrichment, dough production

## Abstract

In this study, the formulation of doughs was investigated using varying percentages of *Agaricus bisporus* flour, with the aim of utilizing mushroom stem fragments, typically considered production waste. The stem residues were collected from a mushroom cultivation facility, cleaned, and washed to remove impurities. The material was then subjected to two different drying methods: conventional dehydration and freeze-drying. After drying, the material was ground to produce mushroom flour. Doughs were formulated with different proportions of this flour and analyzed for texture profile, color, nutritional value, phenolic content, antioxidant activity, and sensory characteristics. The inclusion of mushroom flour resulted in darker doughs, particularly when the flour was obtained through conventional dehydration due to oxidation processes. This substitution also affected texture parameters, leading to increased hardness and reduced elasticity in most treatments compared to the control sample. In addition, cohesiveness progressively decreased from 0.35 in the control to 0.14 in the sample made with 100% dehydrated flour and 0.20 in the sample made with 100% freeze-dried flour, resulting in brittle doughs. The most significant impact on nutritional value was an increase in protein, fat, and dietary fiber levels, reaching values over 5% of crude fiber in the sample to which 50% of dehydrated mushroom flour was added. Additionally, mushroom flours exhibited a high proportion of phenolic compounds, reaching values near 700 mg gallic acid/100 g in the flour from freeze-dried samples and 320 mg gallic acid/100 g in the flour from dehydrated samples. These values reflect a higher content of phenolic compounds in products made with mushroom flours and an increased antioxidant capacity compared to the control sample. Sensory evaluation showed that the texture remained unaffected; however, flavor perception was altered at a 50% mushroom flour substitution. In terms of external appearance, only the 25% freeze-dried mushroom flour formulation was statistically similar to the control, while all other treatments were rated lower.

## 1. Introduction

The growing global demand for food has given rise to concerns about future supply possibilities [1]. This situation is primarily driven by population growth, climate change, wars, and the post-COVID-19 scenario, which has had a negative impact on agriculture [2]. Against this backdrop, the adoption of new technologies is seen as a crucial solution, both for the optimization of production processes and for the reduction of food waste. Approximately one third of all food produced for human consumption is estimated to be wasted, accounting for a total of around 1.3 billion tons per year [3].

Food waste reuse is a growing area of study that seeks to reintroduce by-products of food processing into the creation of new products. Not only does this approach help to minimize waste and reduce environmental impacts but it can also lead to the development of innovative value-added products [4,5,6]. By exploring the potential of this waste, it is possible to make use of nutrients and bioactive components that would otherwise be disposed of, fostering a more circular and sustainable economy [7].

Mushroom production is a branch of agriculture based on sustainability, especially given its use of agricultural waste to generate high-quality foodstuffs [8,9]. Mushrooms are rich in proteins, minerals, and vitamins, being considered low-calorie foods [10]. They have functional properties in the treatment of various diseases [11,12]. A wide variety of bioactive components have been described in mushrooms, granting them a role as hepatoprotective, antiviral, anticancer, or hypocholesterolemic agents. Additionally, their low fat content and high fiber levels may contribute to the prevention of cardiovascular diseases [10,13,14]. Despite its sustainable basis, mushroom production nevertheless generates a significant amount of waste, which is often inadequately disposed of and improperly scrapped, causing a variety of environmental problems [15,16].

The production of *Agaricus bisporus*, one of the most economically significant species worldwide [17,18], generates various types of waste, of which the most notable is the stem residue. This residue represents a loss of from 10 to 13% of the mushroom yield, while, being a part of the fruiting body (fungus), it has similar nutritional values to those found in the rest of the basidiocarp [16,19], potentially being an alternative for food enrichment.

Foods such as pizza are commonly consumed around the world, mainly due to their low cost and simple preparation. However, this type of food is often associated with problems stemming from its high glycemic index [20]. Additionally, traditional varieties of pizza dough are typically poor in fiber and essential nutrients, which can lead to an unbalanced diet if consumed in excess [21,22]. Thus, research on enriching this food through the addition of other products has emerged as a topic of growing interest [21].

The aim of this study is thus to use the stem residue from the production of *Agaricus bisporus* to develop a flour that can be incorporated into the manufacture of doughs. We evaluate the physical, chemical and sensory quality in order to verify the nutritional and technological potential of this by-product as a functional ingredient in food.

## 2. Results and Discussion

### 2.1. Physical Parameters

**Figure 1:**
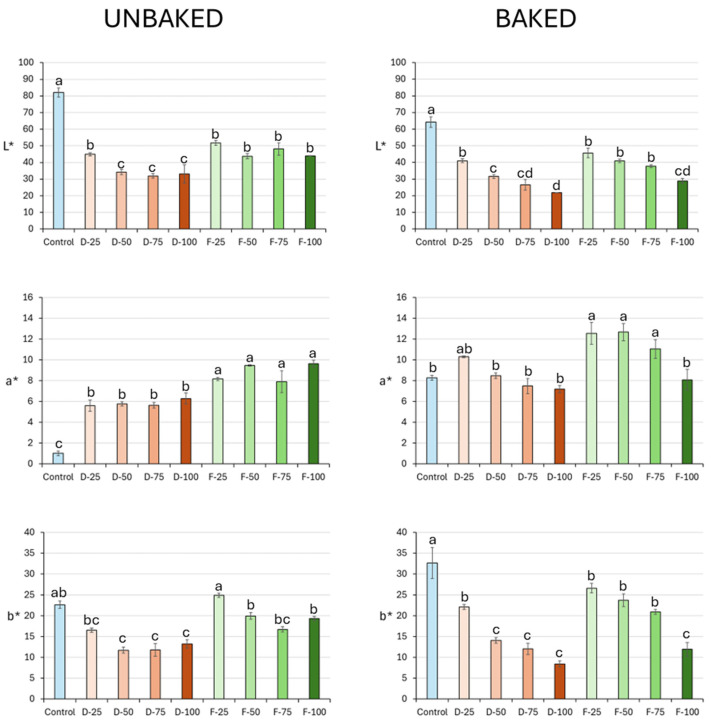
Coloring of the doughs produced with different concentrations of mushroom flour (D: dehydrated; F: freeze-dried), before and after cooking. For each CIELAB parameter, for both unbaked and baked dough, different lower case letters indicate significant differences between the colorimetric parameters of the different samples according to Tukey’s HSD test (*p* < 0.05).

Color was measured in the pizza dough made with increasing concentrations of mushroom flour (25, 50, 75, and 100%). Figure 1 shows the results obtained for the color parameters of both the baked and unbaked doughs with different concentrations of dehydrated and freeze-dried mushroom flour. Color is one of the most important attributes of a food, as it directly impacts consumers’ choice of product [23,24]. Adding mushroom flour to the dough formulation was found to reduce the lightness values, both before and after the baking process. The control, in which mushroom flour was not used, presented the highest lightness values. Comparing the flours obtained by the dehydration and freeze-drying processes, the doughs containing freeze-dried flour showed higher lightness values in both states (raw and cooked), exhibiting differences in coloring according to the drying method used [24,25]. The incorporation of new ingredients in doughs, especially when they contain reducing sugars and proteins, intensifies the Maillard reaction, resulting in alterations in the color of the final product [26,27].

The control sample, followed by the doughs with dehydrated mushroom residues, presented the lowest a* values. As regards the b* parameter (yellow/blue), the control sample yielded the highest mean values, followed by the dehydrated mushroom flour dough, while the doughs containing the freeze-dried flour presented the highest mean scores, both before and after the baking process. The intensity of the yellow is influenced by the addition of mushroom flour, especially after the cooking process, while before baking, a small variation is observed between the flours with 75% and 100% concentrations.

Lightness (L*) and yellowness (b*) are known to be impacted by the use of mushroom flours in the dough formulation [28,29,30]. This effect is primarily the result of the dark color of the mushroom flour following the drying process, with freeze-drying being the method that better preserves the characteristics observed in the control. Meanwhile, the addition of mushroom flour generates higher redness values (a*), with this being the parameter most significantly altered by the inclusion of such flours [28].

A more refined and meaningful analysis of food color changes can be achieved by transforming CIELab color parameters into the Whiteness Index (WI) and Browning Index (BI). Table 1 presents WI and BI measurements for unbaked and baked pizza dough samples. The WI is a key indicator of product refinement and visual consistency, often affecting consumer perceptions and overall product acceptability. Notably, adding mushroom flour significantly decreased WI values in unbaked dough, with a proportional decrease corresponding to the amount of mushroom flour added, particularly when using dehydrated flour. On the other hand, the BI is associated with chemical reactions such as the Maillard reaction or caramelization that occur during thermal processing [31], indicating browning levels which can affect flavor, nutritional content, and shelf life. The BI measurements indicate a proportional increase in browning in unbaked doughs containing mushroom flour. However, these differences are minimized in baked samples, where dehydrated flour samples showed lower BI values than the control.

Table 2 shows the texture parameters of the doughs formulated with different concentrations of dehydrated and freeze-dried mushroom flour. Adding mushroom flour to the dough tends to increase its hardness (N) compared to formulations without such flour [30,32,33]. However, when the dough is composed of 100% mushroom flour, and is not combined with other flours, a reduction in hardness is observed, although the hardness is still higher than that of the standard dough (control). This is caused by the lack of cohesion in the dough, which creates breakage points that result in a lower hardness value. The drying method did not significantly affect the hardness parameters, with the dehydrated and freeze-dried flours presenting statistically similar mean values.

The freeze-dried mushroom flour presented means statistically closer to those of the control for the attributes of cohesiveness and elasticity. However, the greater the flour concentration, the more these parameters were affected; the same occurred with the dehydrated flour. However, the variations observed were not significant, indicating a relatively low impact, as previously evidenced in the literature [33].

The chewiness of the products made with different concentrations of mushroom flour shows no substantive variations compared to traditional products [34]. The mushroom flour obtained by the dehydration method was found to present lower means for chewiness, with all being statistically similar to the control values, except for the 100% concentration, which showed the lowest mean for this parameter. The flours obtained by freeze-drying presented higher mean chewiness values, although these were considerably lower in the case of using 100% mushroom flour in the dough formulation

### 2.2. Proximate Analysis

Significant differences between the wheat flour commonly used in dough production and the mushroom flours obtained through the two drying methods were found (Table 3).

The mushroom flour can be seen to have a higher concentration of proteins, sugar, ashes, and fiber compared to the wheat flour conventionally used in dough preparation. The drying process of mushroom production residues (lower part of the stem), followed by flour preparation, showed few significant differences, the most notable being in total fat content. In this aspect, freeze-drying showed statistically higher means than dehydration, although fat content is not a primary factor in the proximate analysis of mushrooms.

Regarding proximate analysis of the products, like the sensory analysis, this was performed only on the samples in which wheat flour was partially replaced with mushroom flour at 25% and 50%, since the texture analysis showed that a higher percentage of mushroom flour produced harder, less-cohesive doughs that were difficult to handle. As for the nutritional value of the formulated doughs, adding mushroom flour was found to yield a higher content of protein, total fat, and crude fiber compared to the control sample. The two drying methods showed significant statistical differences in almost all parameters, with freeze-drying being the method that showed the greater stability, regardless of the percentage of flour used. The treatment with 25% dehydrated flour presented characteristics closer to the control, except for a significant increase in crude fiber content (Table 4).

The addition of mushroom flour results in an increase in protein content in the formulation of foodstuffs [35,36], as observed in the present study, regardless of the drying method used. The protein matrices are able to retain the starch granules, thus reducing accessibility to enzymatic activity, which modifies the process of starch digestibility [37,38]. Moreover, this increase in protein content may allow this food to be used as a source of daily protein, something that was not previously viable [39].

An slight increase in the concentration of fat was noticed in the mushroom flour, which also helps enrich the food [40]. Although fat is not considered the main nutrient in mushrooms, they have been described as a source of essential fatty acids, since their fat profile is rich in linoleic acid, which can reach levels of up to 68–84% of this fatty acid [41,42]. On the other hand, the most notable contribution of incorporating dehydrated or freeze-dried mushroom flour into pizza dough, in terms of nutritional profile, is the significant increase in dietary fiber. Fiber plays a crucial role in the microbial composition of the human small intestine due to its resistance to digestion [43]. It also helps reduce the glycemic response [38] through its interaction with amylopectin [44]. These aspects evidence the advantages of using mushroom flour to enrich foods.

### 2.3. Phenolic Compounds and Antioxidant Activity

**Figure 2:**
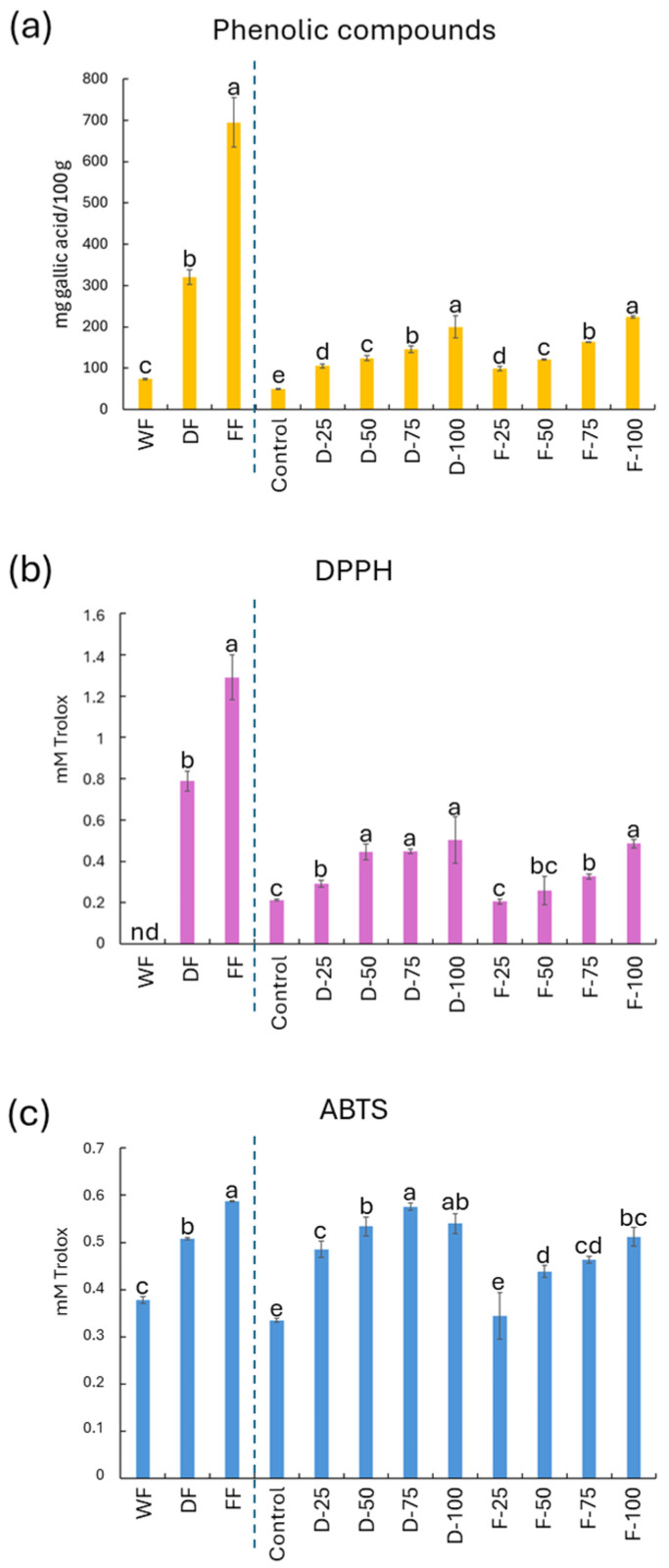
Results for the total phenolic compounds (**a**) expressed in mg of gallic acid/100 g and antioxidant activity measured by DPPH (**b**) and ABTS (**c**) expressed in mM of Trolox equivalent for flours and their baked doughs made with different concentrations of dehydrated and freeze-dried mushroom flour. WF: wheat flour; DF: dehydrated mushroom flour; FF: freeze-dried mushroom flour; Control: cooked wheat dough; D-25: dough cooked with 25% dehydrated mushroom flour; D-50: dough cooked with 50% dehydrated mushroom; D-75: dough cooked with 75% dehydrated mushroom; D-100: dough cooked with 100% dehydrated mushroom; F-25: dough cooked with 25% freeze-dried mushroom flour; F-50: dough cooked with 50% freeze-dried mushroom flour; F-75: dough cooked with 75% freeze-dried mushroom flour; F-100: dough cooked with 25% freeze-dried mushroom flour. For the total phenolic compounds, DPPH, and ABTS, for both flours and baked dough, different lower case letters placed at the top of the bars indicate significant differences between the samples according to Tukey’s HSD test (α < 0.05).

Regarding the content of phenolic compounds present in the flours used, it was found that mushroom flour has a higher amount of these compounds compared to wheat flour, and that the drying method of the mushroom fragments directly affects this availability, with the freeze-drying process promoting a greater increase in such compounds compared to the mushroom residue dried by dehydration (Figure 2).

However, the baking process used for the doughs affected the decrease in total phenolic compounds, mainly due to high temperatures or the oxidation of thermally unstable compounds [38,45]. As expected, the baked doughs containing 100% freeze-dried or dehydrated mushroom flour presented the highest concentration of phenolic compounds (Figure 2). As for the antioxidant activity measured by DPPH (Figure 2), the freeze-dried mushroom flour (F) showed a higher activity compared to the other flours, which decreased in the baking process. As for the dehydrated baked doughs with percentages equal to or higher than 50% (D-50, D-75, and D-100) and F-100, these showed the highest antioxidant capacity. Meanwhile, for the antioxidant activity measured by ABTS (Figure 2), it was similarly observed that the freeze-dried flour was the most outstanding. As for the baked doughs, we observed similar behaviors to those studied with the other methods, with the D-100, D-75, D-50, and F-100 doughs being the most noteworthy.

### 2.4. Sensory Analysis

**Figure 3:**
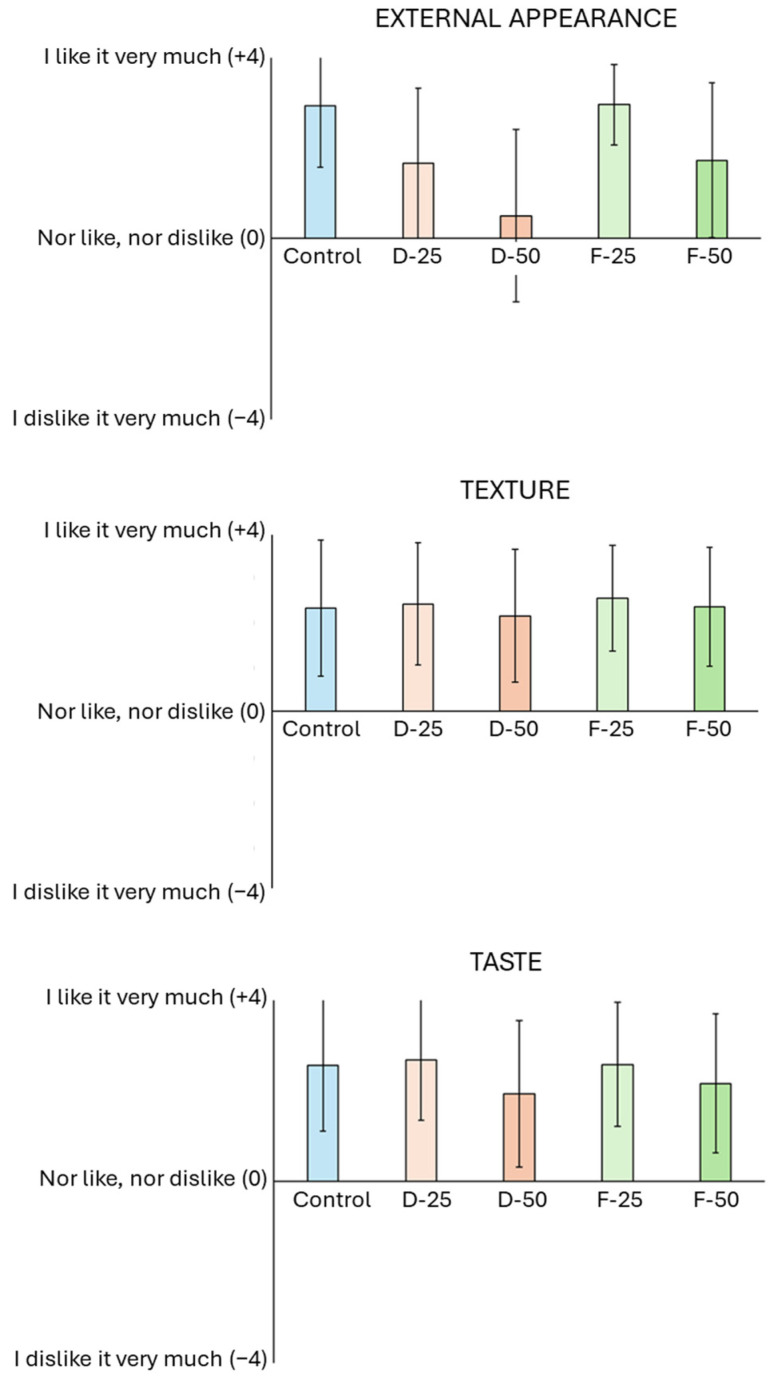
Average values of the sensory analysis regarding external appearance, texture and taste. Control: cooked wheat dough; D-25: dough cooked with 25% dehydrated mushroom flour; D-50: dough cooked with 50% dehydrated mushroom; F-25: dough cooked with 25% freeze-dried mushroom flour; F-50: dough cooked with 50% freeze-dried mushroom flour.

Figure 3 shows the results of the sensory analysis of the pizzas produced using different concentrations of mushroom flour. As regards the external appearance, the pizza doughs made with 25% of freeze-dried mushroom flour present statistically similar values to those of the control sample. However, the additions of higher concentrations compromised the perceived texture, an effect also found in the case of the dehydrated mushroom flour, for which the 50% concentration yielded the lowest mean value in this assessment.

In terms of texture, no significant statistical differences were found between treatments. As regards flavor, meanwhile, the addition of 25% mushroom flour, both in the dehydration and freeze-drying methods, resulted in statistically similar means to the control. However, increasing the flour concentration negatively affected flavor, especially at concentrations of 50% in both drying methods.

Studies have shown that adding large amounts of mushroom flour directly affects product acceptance [46,47,48]. Acceptability is correlated, however, with the type of food produced, such that, depending on the application, the addition of mushroom flour may result in a more positive sensory evaluation compared to the standard product [49].

The way the mushroom fragments were dried also impacts product acceptance, primarily due to the variations in the color of the final foodstuff [50,51]. The color of mushroom flour produced using freeze-drying is closer to that of the control (white). Although the process of collecting and cleaning the mushroom stem residue results in high levels of oxidation due to handling [52], dehydration can increase the respiration rate, leading to enzymatic browning and loss of biochemical compounds, nutrients, flavor, and aroma [53]. The freeze-drying method is known to better preserve essential nutrients and polyphenolic compounds and elements [54].

The predominant flavor of products produced with mushroom flour is an umami taste that is characteristic of mushrooms. However, compounds such as free and soluble sugars and alcohol sugars can impact on the perceived flavor and aroma of the product [55]. High concentrations of mushroom flour may result in a darker color and hence a stronger, more intense flavor, which may have a negative impact on the sensory evaluation [54].

## 3. Materials and Methods

### 3.1. Raw Material

The waste generated in the production of *Agaricus bisporus* was obtained from Mercajúcar, a company located in Villalgordo de Júcar, Albacete, Spain. After collection, the waste underwent a preliminary treatment in which all parts containing traces of the coating layer were removed. The material was then washed under running water until all impurities had been completely eliminated. This process was carried out immediately after collection to avoid the material oxidating.

After washing, the mushroom stem fragments were conditioned in plastic bags, with one part being stored under refrigeration at 5 °C and the other part in the freezer at −20 °C. After 24 h of conditioning, the residue kept under refrigeration was transferred to a dehydrator previously heated to 60 °C, where it remained for 10 h at the same temperature. The other portion, previously stored in the freezer, was submitted to the freeze-drying process at a pressure of 1 Pa and a temperature of −70 °C for 72 h. The dehydrated and freeze-dried mushroom waste were thus obtained.

After drying, the mushroom residues were ground in a blade mill at a speed of 10,000 rpm for 10 s and then sieved using a 1 mm mesh sieve to obtain the mushroom flour. This process was applied to both the dried and freeze-dried mushroom portions.

### 3.2. Dough Production

**Figure 4:**
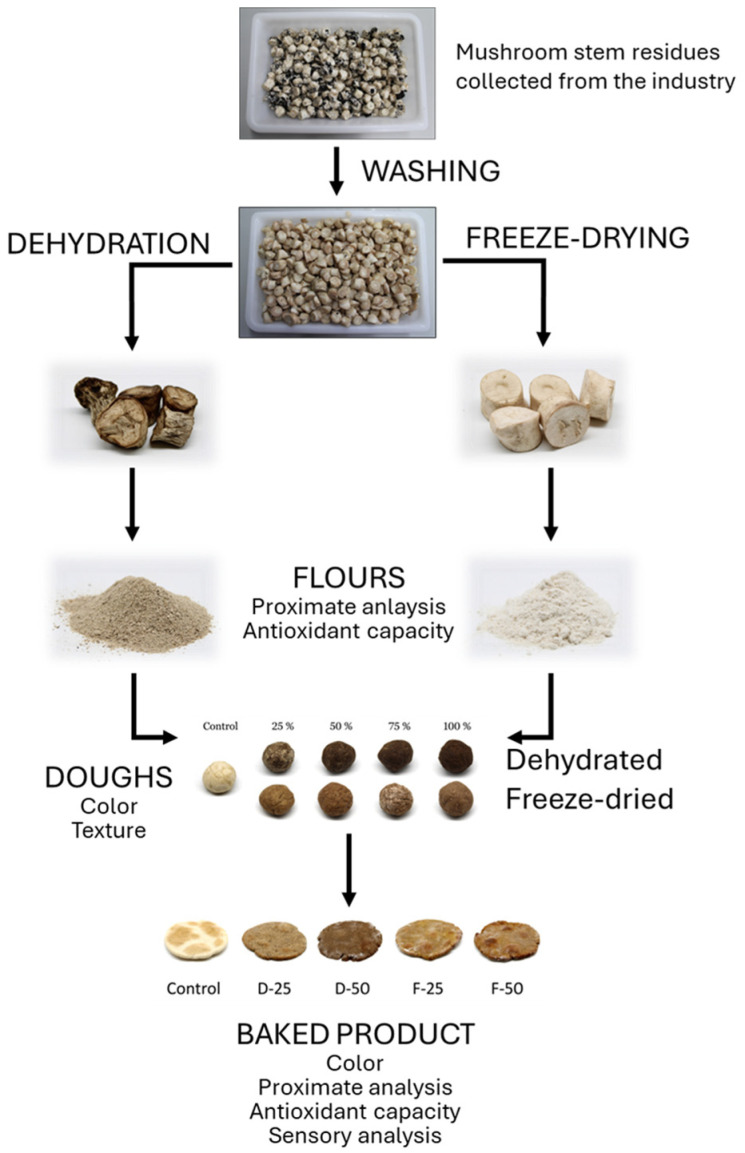
Scheme of the different stages carried out during the process and the analyses performed at each stage.

Wheat flour, mushroom flour, olive oil, and water were used to formulate the different types of doughs in the amounts specified in Table 5. After weighing all the ingredients, the mixture was made manually until the dough reached the appropriate homogeneity and consistency. The physical, chemical, and sensory analyses were then performed (Figure 4).

### 3.3. Physical Analysis

The color of the different dough samples was determined using a Minolta CR-300 colorimeter (Minolta Camera Co., Ltd., Osaka, Japan). The color parameters were measured by reflection on five random areas of the doughs. The illuminant employed was D65. The tristimulus values obtained were used to calculate the CIELab color coordinates L* (relative lightness), a* (red-green component), and b* (yellow-blue component), following the recommendations of the International Commission on Illumination [56]. Three different points of each dough were measured.

CIELab color parameters were used to calculate the Whiteness Index (WI) according to the following formula [57]:

WI=100−100−L*2+a*2+b*212

The Browning Index (BI) was calculated according to the following formula [58]:

$$\mathrm{BI}=\frac{100\left(x-0.31\right)}{0.17}$$

where x is calculated from CIELab parameters according to the following formula:

$$x=\frac{a^{*}+1.75L^{*}}{5.645L^{*}+a^{*}-3.012b^{*}}$$

To measure the texture of the doughs, a texture profile analysis (TPA) was performed using a TA-XT Plus texture analyzer (Stable Micro Systems, Godalming, UK) with a 50 mm diameter probe at 3.3 mm/s^−1^. The analysis was carried out at room temperature, compressing the samples to 60% of their initial height. Finally, hardness, cohesiveness, elasticity, and chewability were determined.

### 3.4. Nutritional Analysis

The nutritional analysis was conducted at the Service for the Analysis and Innovation in Animal Origin Products located on the campus of the University of Extremadura in Cáceres (Spain). We determined the levels of moisture content, protein, total fat, total carbohydrates, crude fiber, and energy value.

The moisture content was determined by placing the sample in an oven at 100 °C for 24 h until a constant weight was reached. Protein content was measured using the Kjeldahl method, multiplying the nitrogen content by a conversion factor of 6.25. The amount of fat was gravimetrically assessed using the filter bag technique after extracting the sample with petroleum ether in an Ankom XT10 (ANKOM Technology, Fairport, NY, USA) extraction system. To measure fiber content, we used the Weende technique adapted for the filter bag, determining the organic residue remaining after digestion with sulfuric acid and sodium hydroxide solutions using an Ankom 220 fiber analyzer (Ankom, Macedon, NY, USA). The carbohydrates were calculated by subtracting the sum of the protein, fat, water, and ash content from the total sample weight. Finally, the energy values were estimated based on protein, fat, and carbohydrate content, using the Atwater factors of 4 kcal/g for protein, 9 kcal/g for fat, and 4 kcal/g for carbohydrate.

### 3.5. Determination of the Total Amount of Phenolic Compounds

To determine the phenolic compounds, 2 g of the dough was weighed, to which 20 mL of methanol was added. This was then shaken for 8 h in an unlit environment. After this shaking, the samples were centrifuged (13,000 rpm, 5 min), with the supernatant then being collected. The samples were kept refrigerated until analysis in an environment without light.

To quantify the phenolic compounds, we used the Folin–Ciocalteu method described by Singleton et al. [59]. To this end, 25 µL of the sample was added to 1500 µL of distilled water and 125 µL of Folin–Ciocalteu reagent, and after 3 min, we added 375 µL sodium carbonate at a concentration of 20% and 475 µL of distilled water. After resting for 2 h in an environment without light, absorbance was measured at 725 nm in a UV-vis spectrophotometer (uniSPEC 4 UV/Vis spectrometer, LLG labware, Meckenheim, Germany). A gallic acid standard was used to prepare the calibration curve from 0 to 3.6 mg/mL. The results are expressed as milligram gallic acid equivalents per milliliter. All the samples were run in triplicate.

### 3.6. Determination of Antioxidant Activity

To determine the antioxidant activity of the different methanolic extracts, we used the ABTS-+ (azino-bis[3-ethylbenzothiazoline-6-sulphonic acid] cation radical) and DPPH- (2, 2-diphenyl-1-picrylhydrazyl radical) methods. The ABTS-+ and DPPH- solutions were prepared following Martinez-Navarro et al. [60]. The calibration line was prepared using the standard trolox solution in methanol at concentrations of 0.05 to 1 mM for ABTS·+. For DPPH-, the calibration line used was from 4000 to 400 mM. The results are expressed as micromole Trolox equivalents per milliliter. All the determinations were conducted in triplicate.

### 3.7. Sensory Analysis

To assess consumer acceptance of the samples, we opted to conduct an affective test. This type of test measures individuals’ subjective reaction to the product. We used the “Test to Measure the Level of Satisfaction”, which uses verbal hedonic scales. The test was conducted at sensory analysis laboratory located at the Higher Technical School of Agricultural and Forestry Engineering and Biotechnology on the Albacete campus of the University of Castilla–La Mancha (UCLM) in Spain.

The samples were identified with randomly selected codes and were evaluated by 103 consumers, who analyzed their external appearance, texture, and flavor. Each hedonic description was rated on a 9-point scale (ranging from −4 = I dislike it immensely, to 0 = I neither like nor dislike it, to +4 = I like immensely) [61]. The samples were served in the form of pizzas produced with doughs containing different concentrations of mushroom stem residue.

### 3.8. Statistical Analysis

All the experimental data obtained were analyzed in three replicate trials, with the results being expressed as mean ± standard deviation, using Excel 365 for Windows (Microsoft Corporation, Redmond, WA, USA). A one-way analysis of variance (ANOVA), together with Tukey’s post hoc test (*p* < 0.05) was performed to determine the significance of the data using SPSS Version 24 for Windows (SPSS INC., Chicago, IL, USA).

## 4. Conclusions

Using *Agaricus bisporus* stem residue for the production of flours and, subsequently, doughs is effective and provides significant benefits in nutritional values, including increases in protein, fat, and fiber. However, the incorporation of this flour affects textural attributes, such as hardness, and alters characteristics of the coloring, resulting in lower lightness and yellow/blue values and higher red/green values, resulting in darker doughs and a more negative sensory evaluation. Nonetheless, the addition of low amounts of flour (up to 25%) did not compromise the sensory evaluation in terms of texture and flavor. Moreover, it increased the antioxidant activity of doughs baked with mushroom flour blends, suggesting that mushroom stems may provide additional functional benefits to flour production.

In addition, future research could focus on evaluating the impact of mushroom flour on specific volume and microscopic morphological features of the dough. Investigating whether the addition of mushroom flour influences fermentation processes and the resulting dough structure could provide valuable insights into optimizing formulations for texture and volume. Furthermore, conducting microscopic analyses on internal structures may reveal interactions at a cellular level that contribute to the unique textural and sensory properties observed. Expanding on these aspects would deepen our understanding and guide further improvements in the production of mushroom flour-enriched doughs.

## Figures and Tables

**Table 1:** Whiteness Index (WI) and Browning Index (BI) of the different reformulations of unbaked and baked dough made with mushroom flour.

		Control	D25	D50	D75	D100	F25	F50	F75	F100
WI	Unbaked	71.00 ^a^ ± 2.31	42.23 ^b^ ± 0.88	32.97 ^c^ ± 1.51	30.71 ^c^ ± 1.03	31.54 ^c^ ± 5.41	45.11 ^b^ ± 1.08	39.64 ^b^ ± 1.27	44.90 ^b^ ± 3.80	39.98 ^b^ ± 0.23
	Baked	51.10 ^a^ ± 5.08	36.18 ^b^ ± 0.87	29.58 ^c^ ± 1.07	25.18 ^c^ ± 2.89	21.02 ^d^ ± 0.08	38.18 ^b^ ± 2.21	35.14 ^b^ ± 0.50	33.36 ^b^ ± 0.73	27.38 ^c^ ± 1.31
BI	Unbaked	32.64 ^c^ ± 2.80	54.24 ^b^ ± 2.39	53.75 ^b^ ± 0.89	58.38 ^b^ ± 5.23	74.34 ^a^ ± 2.10	75.58 ^a^ ± 1.30	75.32 ^a^ ± 1.87	74.70 ^a^ ± 8.16	72.87 ^a^ ± 2.51
	Baked	87.39 ^b^ ± 7.10	93.14 ^b^ ± 0.78	77.67 ^c^ ± 1.17	79.74 ^c^ ± 1.40	72.09 ^c^ ± 5.75	104.02 ^a^ ± 5.39	105.30 ^a^ ± 3.03	99.38 ^a^ ± 1.78	77.28 ^c^ ± 4.79

Control: wheat flour dough; D-25: dough with 25% dehydrated mushroom flour; D-50: dough with 50% dehydrated mushroom flour; D-75: dough with 75% dehydrated mushroom flour; D-100: dough with 100% dehydrated mushroom flour; F-25: dough with 25% freeze-dried mushroom flour; F-50: dough with 50% freeze-dried mushroom flour; F-75: dough with 75% freeze-dried mushroom flour; F-100: dough with 100% freeze-dried mushroom flour. Different lower case letters in the same line indicate significant differences between the different samples according to Tukey’s HSD test (*p* < 0.05).

**Table 2:** Results for the texture parameters of the different reformulations of dough made with mushroom flour.

Sample	Hardness (N)	Cohesiveness	Elasticity	Adhesiveness	Chewiness
Control	44.14 ^d^ ± 7.95	0.35 ^a^ ± 0.03	0.23 ^a^ ± 0.02	−0.42 ^c^ ± 0.04	3.61 ^b^ ± 0.3
D-25	69.81 ^c^ ± 8.50	0.28 ^b^ ± 0.02	0.19 ^ab^ ± 0.03	−0.56 ^c^ ± 0.03	3.64 ^b^ ± 0.2
D-50	105.74 ^b^ ± 6.15	0.24 ^bc^ ± 0.02	0.14 ^b^ ± 0.04	−0.7 ^c^ ± 0.05	3.55 ^b^ ± 0.2
D-75	130.33 ^a^ ± 11.33	0.19 ^c^ ± 0.01	0.12 ^b^ ± 0.04	−0.11 ^c^ ± 0.05	3.01 ^bc^ ± 0.1
D-100	54.37 ^d^ ± 9.97	0.14 ^d^ ± 0.03	0.13 ^b^ ± 0.06	−0.07 ^c^ ± 0.03	0.98 ^d^ ± 0.4
F-25	71.73 ^c^ ± 7.33	0.30 ^ab^ ± 0.05	0.20 ^ab^ ± 0.07	−3.50 ^a^ ± 0.01	4.25 ^ab^ ± 0.4
F-50	106.06 ^b^ ± 5.41	0.27 ^b^± 0.06	0.17 ^b^ ± 0.01	−3.52 ^a^ ± 0.02	5.00 ^a^ ± 0.5
F-75	135.33 ^a^ ± 12.81	0.23 ^bc^ ± 0.07	0.14 ^b^ ± 0.08	−3.35 ^a^ ± 0.06	4.34 ^ab^ ± 0.6
F-100	97.48 ^bc^ ± 10.12	0.20 ^c^ ± 0.06	0.12 ^b^ ± 0.02	−2.44 ^b^ ± 0.07	2.44 ^c^ ± 0.7

Control: wheat flour dough; D-25: dough with 25% dehydrated mushroom flour; D-50: dough with 50% dehydrated mushroom flour; D-75: dough with 75% dehydrated mushroom flour; D-100: dough with 100% dehydrated mushroom flour; F-25: dough with 25% freeze-dried mushroom flour; F-50: dough with 50% freeze-dried mushroom flour; F-75: dough with 75% freeze-dried mushroom flour; F-100: dough with 100% freeze-dried mushroom flour. Different lower case letters indicate significant differences between the texture parameters of the different samples according to Tukey’s HSD test (*p* < 0.05).

**Table 3:** Result of the nutritional analysis of the different flours used to produce the dough.

Sample	Protein	Total Fat	Saturated Fat	Total Carbohydrates	Sugar	Ash	Crude Fiber	Energy (Kcal)
Wheat flour	11.6 ^b^ ± 0.7	1.2 ^ab^ ± 0.4	0.7 ± 0.05	77.3 ^a^ ± 10.2	2.2 ^b^ ± 0.06	0.5 ^b^ ± 0.01	0.8 ^b^ ± 0.1	366 ^a^ ± 14.4
DF	16.7 ^a^ ± 0.6	0.7 ^b^ ± 0.1	0.4 ± 0.03	67.6 ^b^ ± 8.8	3.2 ^a^ ± 0.05	3.2 ^a^ ± 0.05	9.9 ^a^ ± 0.8	344 ^b^ ± 11.8
FF	17.4 ^a^ ± 0.5	1.8 ^a^ ± 0.7	0.6 ± 0.02	68.5 ^b^ ± 11.7	2.7 ^ab^ ± 0.07	3.1 ^a^ ± 0.03	8.8 ^a^ ± 0.6	359 ^ab^ ± 10.2

DF: dehydrated mushroom flour; FF: freeze-dried mushroom flour. Different lower case letters indicate significant differences between the nutritional parameters of the different samples according to Tukey’s HSD test (*p* < 0.05).

**Table 4:** Results of the nutritional analysis of the different formulations of dough prepared with mushroom flour.

Sample	Protein	Total Fat	Saturated Fat	Total Carbohydrates	Sugar	Ash	Crude Fiber	Energy (Kcal)
Control	9.3 ^b^ ± 0.3	3.9 ^b^ ± 0.1	1.1 ^b^ ± 0.02	62.5 ^a^ ± 7.3	0.6 ^ab^ ± 0.02	0.4 ^c^ ± 0.01	1.2 ^d^ ± 0.3	323 ± 7.3
D-25	9.6 ^ab^ ± 0.4	4.2 ^b^ ± 0.4	1.2 ^b^ ± 0.03	60.1 ^ab^ ± 6.5	1.1 ^a^ ± 0.03	0.6 ^c^ ± 0.04	4.3 ^bc^ ± 0.6	316 ± 11.4
D-50	11.6 ^a^ ± 0.4	6.4 ^a^ ± 0.5	1.6 ^a^ ± 0.05	57.5 ^b^ ± 4.9	1.1 ^a^ ± 0.04	1.4 ^a^ ± 0.05	5.2 ^a^ ± 0.5	334 ± 9.2
F-25	11.6 ^a^ ± 0.5	5.9 ^a^ ± 0.5	1.5 ^a^ ± 0.06	58.0 ^b^ ± 5.3	0.2 ^b^ ± 0.01	1.4 ^a^ ± 0.03	3.3 ^c^ ± 0.5	326 ± 12.6
F-50	10.4 ^ab^ ± 0.2	5.8 ^a^ ± 0.2	1.7 ^a^ ± 0.09	56.9 ^b^ ± 9.6	0.7 ^ab^ ± 0.01	1.1 ^b^ ± 0.02	3.5 ^c^ ± 0.7	321 ± 12.9

Control: wheat flour dough; D-25: dough with 25% dehydrated mushroom flour; D-50: dough with 50% dehydrated mushroom flour; F-25: dough with 25% freeze-dried mushroom flour; F-50: dough with 50% freeze-dried mushroom flour. Different lower case letters indicate significant differences between the nutritional parameters of the different samples according to Tukey’s HSD test (*p* < 0.05).

**Table 5:** Formulation of ingredients to make the doughs, composed of different percentages of dehydrated and freeze-dried mushroom flour.

Code	Sample	Wheat Flour (g)	Mushroom Flour (g)	Oil (g)	Water (g)
C	Control	50	0	4	27.5
D-25	Dehydrated 25	37.5	12.5	4	27.5
D-50	Dehydrated 50	25	25	4	27.5
D-75	Dehydrated 75	12.5	37.5	4	27.5
D-100	Dehydrated 100	0	50	4	32.5
F-25	Freeze-dried 25	37.5	12.5	4	27.5
F-50	Freeze-dried 50	25	25	4	27.5
F-75	Freeze-dried 75	12.5	37.5	4	27.5
F-100	Freeze-dried 100	0	50	4	32.5

## Data Availability

The data presented in this study are available on request from the corresponding author.
